# CIRRHOTIC PATIENTS WITH ACUTE KIDNEY INJURY (AKI) HAVE HIGHER MORTALITY AFTER ABDOMINAL HERNIA SURGERY

**DOI:** 10.1590/0102-672020210002e1622

**Published:** 2021-12-17

**Authors:** Liliana DUCATTI, Luciana B. P. HADDAD, Alberto MEYER, Lucas S. NACIF, Rubens M. ARANTES, Rodrigo B. MARTINO, Vinicius ROCHA-SANTOS, Daniel R. WAISBERG, Rafael S. PINHEIRO, Luiz A.C. D´ALBUQUERQUE, Wellington ANDRAUS

**Affiliations:** 1 Department of Gastroenterology, University Hospital, School of Medicine, University of São Paulo - USP, São Paulo, SP, Brazil

**Keywords:** Liver cirrhosis, Herniorrhaphy, Hernia, Acute kidney injury, Ascites, Cirrose hepática, Herniorrafia, Hérnia, Injuria renal aguda, Ascite

## Abstract

**Background:**

The incidence of abdominal hernia in cirrhotic patients is as higher as 20%; in cases of major ascites the incidence may increase up to 40%. One of the main and most serious complications in cirrhotic postoperative period (PO) is acute kidney injury (AKI).

**Aim:**

To analyze the renal function of cirrhotic patients undergoing to hernia surgery and evaluate the factors related to AKI.

**Methods:**

Follow-up of 174 cirrhotic patients who underwent hernia surgery. Laboratory tests including the renal function were collected in the PO.AKI was defined based on the consensus of the ascite´s club. **They were divided into two groups: with (AKI PO) and without AKI** .

**Results:**

All 174 patients were enrolled and AKI occurred in 58 (34.9%). In the AKI PO group, 74.1% had emergency surgery, whereas in the group without AKI PO it was only 34.6%.In the group with AKI PO, 90.4% presented complications, whereas in the group without AKI PO they occurred only in 29.9%. Variables age, baseline MELD, baseline creatinine, creatinine in immediate postoperative (POI), AKI and the presence of ascites were statistically significant for survival.

**Conclusions:**

There is association between AKI PO and emergency surgery and, also, between AKI PO and complications after surgery. The factors related to higher occurrence were initial MELD, basal Cr, Cr POI. The patients with postoperative AKI had a higher rate of complications and higher mortality.

## RESUMO

**Racional:**

A incidência de hérnia abdominal em pacientes cirróticos é elevada, em torno de 20%. Em casos de ascite volumosa, a incidência atinge valores até 40%. Uma das principais e mais graves complicações no pós-operatório de correção de hérnias de pacientes cirróticos é a insuficiência renal aguda (IRA).

**Objetivo:**

Analisar a função renal de pacientes cirróticos submetidos a herniorrafias, comparando aqueles que apresentavam IRA pós-operatório com os demais, para determinar os fatores relacionados à sua ocorrência.

**Método:**

Seguimento de pacientes cirróticos submetidos à cirurgia de hérnia entre 2001 e 2014 no Serviço de Transplante de Fígado. Foram coletados exames laboratoriais para avaliar a função renal no pós-operatório rotineiramente. A IRA foi definida com base no consenso do clube da ascite em 2015.

**Resultados:**

Dos 174 pacientes incluídos, ocorreu IRA em 58 pacientes (34,9%). Houve diferença entre grupos para as seguintes variáveis: MELD inicial, creatinina basal e creatinina, o grupo com IRA apresentou medias superiores ao grupo que não apresentou IRA. No grupo IRA PO, 74,1% das cirurgias, foram realizadas em caráter de emergência, enquanto que no grupo sem IRA no pós-operatório, 34,6%. No grupo IRA, 90,4% dos indivíduos apresentaram complicações no pós-operatório, enquanto no grupo sem IRA, 29,9%. As variáveis idade, MELD inicial, creatinina basal e creatinina no pós-operatório inicial foram estatisticamente significantes na análise de sobrevida.

**Conclusões:**

Existe uma associação entre IRA pós-operatória e cirurgia de emergência e IRA pós-operatóri e complicações pós-operatórias. Os fatores relacionados à maior ocorrência de IRA em pacientes cirróticos submetidos à cirurgia de hérnia são o MELD inicial, creatinina basal, creatinina pós-operatória inicial. O preparo de pacientes cirróticos com hérnia abdominal antes de procedimentos cirúrgicos deve ocorrer sistematicamente, pois apresentam alta incidência de IRA pós-operatória.

## INTRODUCTION

Cirrhosis represents a late stage of progressive hepatic fibrosis characterized by distortion of the hepatic structure and the formation of regenerative nodules. It´s physiopathology involves progressive hepatic lesions resulting in portal hypertension and decompensation, including ascites, spontaneous bacterial peritonitis, hepatic encephalopathy, variceal bleeding, hepatorenal syndrome^12^, hepatocellular carcinoma^17^ and colon dysfunction^6^. It is the 8^th^ most common cause of mortality in the United States and 13^th^ worldwide, with 45.6% increase in mortality. It is generally considered irreversible in its more advanced stages, for which the only definitive treatment is liver transplantation^7^.

One of the principal and most serious complications of cirrhosis is acute kidney injury (AKI)^11^. Its appearance at any phase of cirrhosis is associated with greater morbimortality. Often, its progression leads the patient to renal replacement therapy (dialysis), either temporarily or permanently. In the long term, kidney transplantation may be necessary^13^.Almost 20% of patients with cirrhosis will develop AKI over the course of hospitalization, with mortality rates as high as 50-90%(3). Over 60% of all cases of AKI in patients with cirrhosis are attributable to pre-renal factors, such as hypovolemia, hypoperfusion, massive renal vasoconstriction by systemic arterial and splanchnic vasodilation^5^.

Many recent studies have assessed the incidence of AKI, its evolution and treatment in patients with cirrhosis^19^. There is a lack, however, in the literature assessing the incidence of AKI in patients with cirrhosis, either with or without ascites, who have been submitted to surgical procedures.

The objective of this study was to analyze the renal function of patients with cirrhosis submitted to a correction of abdominal wall hernias. Furthermore, it aimed to compare patients presenting acute kidney injury with others, to determine risk factors for its occurrence. 

## METHODS

This study was approved by the Scientific Ethic Committee of the Department of Gastroenterology School of Medicine, University of São Paulo, São Paulo, SP, Brazil) and is in agreement with all requirements of the Helsinki Declaration, 1975.

This is a retrospective cohort between 2001 and 2014, with analysis of medical records between 2001 and 2009 and analytical, longitudinal collection of prospective data from 2009 to 2014. Patients over the age of 16 with cirrhosis and abdominal hernias who underwent surgical correction of the hernias were included. These patients were monitored during hospitalization and in outpatient clinic during the postoperative phase.

The ones submitted to elective and emergency surgery were assessed and followed. The indications for emergency surgery were: hernial strangulation, painful irreducible hernia, skin perforation with extravasation of ascitic fluid or ischemic ulceration of the skin. These cases were submitted to emergency herniorrhaphy. The following demographic and clinical variables of the patients were assessed: gender, age, BMI (body mass index), etiology of cirrhosis, Child score, MELD score, patient’s inclusion on the liver transplantation waiting list, ascites volume, diabetes, presence of TIPS(transjugular intrahepatic portosystemic shunt), types of hernia, and the presence of previous complications from hernias. The data were collected prior to surgery, in the pre-operative phase, or at hospitalization in emergency cases.

The laboratory tests were collected immediately before the surgery were: creatinine, INR (international normalized ratio), total bilirubin, sodium. Ten grams of human albumin were intravenously administered per liter of ascitic fluid removed during surgery. In cases of voluminous ascites in the postoperative phase, a relief parecentesis was carried out where there was tense ascites, and always with albumin expansion when the volume was over 5 l, in a dose of 10g of albumin per liter removed^10^.

Patients were divided into two groups: patients with AKI and without AKI after surgery.

Laboratorial tests were collected in the immediate postoperative phase, on the 1^st^, 3^rd^and 5^th^postoperative days: creatinine, INR, total bilirubin and sodium. The patients not displaying complications were discharged on the 5^th^postoperative day. In cases where there were significant alterations in results, or a clinical complication, the exams were carried out daily. Diuretics were not introduced in the early post-operative phase for these patients. It was done only at hospital discharge, or after the 5^th^day without AKI signs. The patients who developed AKI received treatment with albumin, with initial doses of 10g administered three times per day^10^ ^,^ ^18^. Patients who showed no improvement of renal function after albumin treatment received terlipressin in initial doses of 0.5mg, four times per day.

### Statistical analysis

Quantitative variables are presented as mean, median, standard-deviation, range, interquartile range. The qualitative variables are presented as absolute frequency and percentage. To verify the behavior of the variables in each group across time, ANOVA was used for repeated, non-parametric measures. To verify the differences between groups of quantitative variables, the Kolmogorov-Smirnov was first used to assess normality. When the supposition of normality was not rejected, the Student’s t-test was used, if it was, the Wilcoxon-Mann-Whitney was used. Fisher’s exact test was used to assess the relation between qualitative variables. Kaplan-Meier estimates were used to create the survival curves for each group, and the log-rank test to assess whether the variables were different. Cox’s model was used to verify which quantitative variables were associated with mortality. The level of significance adopted was 5%. 

## RESULTS

Were included 174 patients who underwent procedures to correct hernias of the abdominal wall. The mean follow-up time was 2.58 years, with a median of 2.3 years (8-2241 days). The mean age was 53.71±11.66 years (Table 1). In the population, 135 patients were male, corresponding to 77.6%.

**Table 1:** Clinical and laboratory characteristics of the 174 patients operated for abdominal wall hernias

Variable	Mean	(1)
Age	53.71	11.66
BMI	25.78	4.31
Time	1238.06	1104.06
MELD Initial	13.00	6.00
Cr basal	1.17	0.69
Cr POI	1.27	1.84
Cr1	1.44	1.01
Cr3	1.52	1.30
Cr5	1.40	1.13
Cr10	1.44	1.01

BMI=body mass index; MELD=Model for End-Stage Liver DiseaseCr=creatinine; PO=postoperative day;POI=1^st^ PO; Cr 1=creatinine on 1^st^PO; Cr 3creatinine on the 3^rd^PO; Cr 5=creatinine on the 5^th^PO; Cr 10=creatinine on the 10^th^PO.(1) internalization (standard-deviation)

The majority of patients were Child B (53.4%), followed by Child A (25.3%) and C (19%). 

From the total of patients who underwent surgery 27.3% had light ascites; 22.4% moderate; 13.2% voluminous; and 17.8% did not have. Of the patients with ascites, 47.9% had previously undergone paracentesis. 

With regards to hernia type, 126 patients had umbilical hernias (72.4%). This was the most common hernia in the evaluated population, and 58 had inguinal hernias (33.3%). Of these, 14 (8%) were bilateral and 10 (6.0%) had incisional hernias. 

Among the entire group (n=174), 88 underwent elective and 83 (48.5%) emergency surgery. In total, 78 (48.5%) had surgical complications. 

### Comparison between patients with and without postoperative AKI

A total of 58 patients (34.9%) had postoperative AKI. Table 2 shows the base creatinine and creatinine of each group in the immediate postoperative phase, and on the 1^st^, 3^rd^and 5^th^postoperative days.

**Table 2:** AKI PO and base creatinine, POI, 1^st^, 3^rd^ and 5^th^ PO

AKI PO	Creatinine	Mean	Median	Standard deviation	Min	Max	1^st^Quartile	3^rd^Quartile
No	Cr base	1.019	0.920	0.579	0.510	6.080	0.770	1.100
	Cr FPD	1.039	0.910	0.708	0.000	6.560	0.920	1.690
	Cr1	1.170	1.070	0.916	0.000	7.710	0.815	1.100
	Cr3	1.149	1.000	1.338	0.000	11.340	1.070	2.170
	Cr5	0.841	0.820	0.726	0.000	2.460	0.800	1.400
Yes	Cr base	1.456	1.240	0.804	0.560	4.620	1.205	2.400
	Cr FPD	1.692	1.350	0.928	0.580	4.720	0.690	1.410
	Cr1	1.984	1.960	0.960	0.000	5.110	1.445	2.555
	Cr3	2.198	2.100	0.918	0.560	4.400	0.000	1.320
	Cr5	2.229	2.105	1.122	0.000	5.400	1.330	2.910

AKI=acute kidney injury; PO=postoperative day; Cr=creatinine; FPD=1^st^PO; Cr 1=creatinine in 1^st^PO; Cr 3=creatinine in 3^rd^PO; Cr 5=creatinine in 5^th^PO; Cr 10=creatinine in 10^th^PO; POI=immediate postoperative

**Figure 1:**
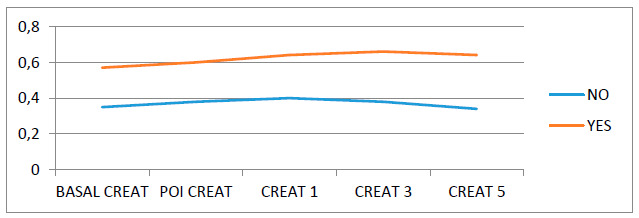
Postoperative creatinine in cirrhotic patients who underwent herniorraphies: A) red line with AKI; B) black line without

The interaction was statistically significant. Creatinine behaved differently over time, depending on the group. In the group that had postoperative AKI, there was an increase in creatinine over time, while in the group that did not have postoperative AKI there was a small increase, followed by a decrease (after Cr3, Figure 1).

CREAT=creatinine;POI=immediate postoperative; Anova for non-parametric repeated measures; red line patients with AKI; black line without AKI.

### Comparison of groups (postoperative AKI)

The variables were tested in relation to the presence of postoperative AKI. There was a significant difference between the groups in the following variables: initial MELD, base creatinine and immediate postoperative creatinine (Table 3). In all variables the postoperative AKI group had higher means than the group without postoperative AKI. There was no difference observed in age or BMI. 

**Table 3:** Variables for the AKI group

Variable	AKI PO	Mean	Standard deviation	p (1)
Age	No	53.86	11.54	0.7720
	Yes	52.79	11.87	
BMI	No	25.83	3.99	0.5999
	Yes	25.47	4.69	
Initial MELD	No	12.00	4.00	0.0001
	Yes	14.00	4.00	
Cr base	No	1.02	0.58	<0.0001
	Yes	1.46	0.80	
Cr POI	No	1.04	0.71	<0.0001
	Yes	1.69	0.93	

BMI=body mass index; MELD=Model For End-Stage Liver Disease;Cr=creatinine; POI=immediate postoperative. (1) Wilcoxon-Mann-Whitney test.

There was no relation between etiology and postoperative AKI. 

In the AKI group, 37.9% had moderate ascites, while in the group without AKI, only 15.7% did (p=0.0001), indicating an association between the degree of ascites and postoperative AKI. 

In the AKI group, 69.1% had undergone paracentesis, while, in the group without AKI, only 38.1% had (p=0.0002). Moreover, in the AKI group, a larger number of patients underwent emergency surgery (Table 4). 

**Table 4:** Postoperative AKI, elective surgery compared with emergency surgery and complications for patients with cirrhosis submitted to hernial correction

Surgery		PO AKI				Total	
		No		Yes			
		n	%	n	%	n	%
	Elective	70	65.4%	15	25.9%	85	51.5%
	Emergency	37	34.6%	43	74.1%	80	48.5%
	Total	107	100.0%	58	100.0%	165	100.0%
PO complication		PO AKI				Total	
		No		Yes			
		n	%	n	%	n	%
	No	68	70.1%	5	9.6%	73	49.0%
	Yes	29	29.9%	47	90.4%	76	51.0%
	Total	97	100.0%	52	100.0%	149	100.0%

p<0.0001; AKI=acute kidney injury; PO=postoperative

In patients with postoperative AKI, there was a larger proportion of individuals with postoperative complications (Table 4). 

### Analysis of survival

The variables were analyzed in relation to patient survival. The presence of ascites, surgery, surgical complications and postoperative AKI were significantly related to survival (Table 5). 

**TABLE 5:** Analysis of factors related to patient survival of patients with cirrhosis submitted to hernial correction - qualitative variables

Variable	p (1)
Ascites	0.0018
Surgery	<0.0001
Surgicalcomplications	0.0025
PO AKI	0.0001

AKI=acute kidney injury; PO=postoperative. (1) Log-rank test to determine whether or not the survival curves are different by Kaplan-Meier estimates.

The variables age, initial MELD, base creatinine and creatinine in the immediate postoperative phase were statistically significant, meaning that the risk ratio is different to 1. For each year of age, the risk of mortality increases by 1.0328 (or increases by 3.28%). In base creatinine, for each increase in unit, the risk of mortality increases by 1.514 (Table 6).

**Table 6:** Analysis of factors related to patient survival of patients with cirrhosis submitted to hernial correction - quantitative variables

Variable	Risk ratio	CI (95,0%) of RR		p (1)
		Minlim	Max lim	
Age	1.0328	1.0079	1.0583	0.0096
BMI	0.9652	0.9006	1.0345	0.3167
Initial MELD	1.0942	1.0391	1.1522	0.0006
Base Cr	1.5140	1.2372	1.8529	0.0001
Cr POI	1.3795	1.1402	1.6691	0.0009

BMI=body mass index; MELD=Model for End-Stage Liver Disease; Cr=creatinine; POI=immediate postoperative. (1) Univariate analysis of risk ratio (RR).

Three different models for multivariate analysis were carried out using Cox models. The variables in each model were: model 1: age, initial MELD and base creatinine; model 2: age, initial MELD and IPO creatinine; model 3: age, initial MELD and creatinine delta (difference between IPO creatinine and base creatinine).

In model 1, the variable base creatinine was not statistically significantly related. The other variables had a risk ratio greater than 1, indicating that they were risk factors for mortality (Table 7). In model 2,immediate postoperative creatinine was not statistically significant. The other two factors again had a risk ratio greater than 1, indicating that they are risk factors for mortality (Table 7). In model 3,delta creatinine was not statistically significant. The other two factors again had a risk ratio greater than 1, indicating that they are risk factors for mortality (Table 7). 

**Table 7:** Multivariate analysis of factors related to survival of patients with cirrhosis who underwent hernial correction - Model 1, Model 2 and Model 3

Model 1	Variable	RR	CI (95.0%) of RR		p
			Min limit	Max limit	
	Age	1.0430	1.0152	1.0715	0.0023
	Initial MELD	1.1041	1.0337	1.1793	0.0032
	Base Cr	1.1913	0.9170	1.5478	0.1899
Model 2	Variable	RR	CI (95,0%) of RR		p
			Min limit	Max limit	
	Age	1.0356	1.0083	1.0637	0.0102
	Initial MELD	1.0932	1.0244	1.1667	0.0072
	Cr POI	1.1633	0.9225	1.4668	0.2011
Model 3	Variable	HR	IC (95,0%) HR		p
			Inflimit	Suplimit	
	Age	1.0391	1.0109	1.0681	0.0063
	Initial MELD	1.1091	1.0473	1.1744	0.0004
	Delta Cr	1.4849	0.8181	2.6952	0.1937

MELD=Model for End-Stage Liver Disease; Cr=creatinine; POI=immediate postoperative

## DISCUSSION

This study of 174 patients represents the largest renal function behavior analysis of patients with cirrhosis undergoing abdominal hernia correction operated on in the same service. This is the first study that assesses kidney function in the postoperative phase and risk factors for renal damage. The main prognostic factors related to survival were: the presence of ascites, umbilical hernia, inguinal hernia, type of surgery, postoperative complications and postoperative AKI.

There is a lack of perioperative studies that show the assessment and evolution of the results of surgery in patients with cirrhosis and complications(9), and this study aimed to identify risk factors for AKI in this population.

This surgery is carried out more frequently today than in the past, in part due to the increased long term survival of patients with cirrhosis. Recent studies have concentrated on estimating perioperative risk in patients with liver disease^15^. Hemodynamic instability in the perioperative period can worsen hepatic function in patients with cirrhosis^8^. The operative risk is correlated with the severity of liver disease and the type of surgical procedure. Detailed evaluation is necessary prior to elective surgery in these patients. The estimated perioperative mortality is unknown due to the retrospective nature and selection bias in published clinical studies. Child-Pugh classification, and particularly MELD score give reasonable estimates for perioperative mortality risk, but do not supplant the need for careful preoperative preparation and postoperative follow up, such as early detection of complications, which is essential to improve outcomes^2^.

The incidence of complications in this study was 44.8%, with ascites leakage through the operative wound, hematoma of the operative wound and skin dehiscence as the most common. This reflects the high rate of complications in this group of patients, as shown in the literature^2^.

The majority of hernias operated in this study were umbilical, representing 72.4% of procedures. This prevalence in patients with cirrhosis is also reflected in the literature^1^.

Patients with cirrhosis have a high incidence of abdominal wall hernias, and following surgical correction, have a higher morbimortality. The ideal surgical strategy, as well as time to correct abdominal wall hernias is controversial^14^ ^,^ ^16^. The advantage of having this knowledge would be to predict which patients are at elevated risk of developing postoperative AKI. This would allow a choice wherever possible, of better preparation before the operation, or attempting a prior intervention or treatment to prevent hernia’s appearance.

A cohort study by Andraus et al.^1^ evaluated 67 patients with cirrhosis submitted to hernial correction. The median MELD score was 14 (6-24). Emergency surgery was carried out in 34 patients due to hernial rupture (n=13), incarceration (n=10), strangulation (n=4) and skin necrosis or ulceration (n=7). Elective surgery was carried out in 27 cases. After a multivariate analysis, emergency surgery (OR 7.31; p=0.017) and Child Pugh C (OR 4.54; p=0.037) were risk factors for serious complications. Furthermore, emergency surgery was an independent risk factor for 30 days mortality (OR 10.83; p=0.028). The risk of morbidity and mortality is associated with emergency surgery in patients with advanced cirrhosis.

As mentioned, majority of patients in this study was Child B (53.4%), followed by Child A (25.3%) and C (19%) and the MELD score varied between 6 and 24. 

Kidney dysfunction in cirrhosis is mainly related to the development of AKI, caused by acute hemodynamic alterations. It´s incidence increases according to the presence of other associated risk factors that can cause alteration to kidney function, such as diabetes, viral hepatitis, among others. AKI is defined as an increase in serum creatinine of 0.3mg/dl in <48 h or of 50% of base creatinine over the past three months, without defining a threshold of final serum creatinine. Stages 1, 2 and 3 of AKI are defined as 150%, 200% and 300% of base serum creatinine, respectively, which allows assessment of the progression of AKI^20^.

Among the sample (n=174)58 patients (34.9%) had postoperative AKI, while 108 (65.1%) did not. The factors related with AKI are initial MELD, base Cr, immediate postoperative Cr.

The main limitation of this study is being retrospective, meaning that it was not possible to verify results by comparing intervention with no intervention.

Abdominal wall hernia is a common complication for patients with cirrhosis. Herniorrhaphy presents a higher morbimortality when compared with patients without cirrhosis. The presence of AKI in the postoperative is a frequent event and should be prevented when possible and treated adequately, in order to reduce mortality in this population.

Preoperative preparation with adequate volume expansion to replace ascites evacuation, careful hydration, an elective surgical approach, rigorous controls of hydration balance in the intra and postoperative phases are useful and effective measures to prevent AKI in patients with cirrhosis. As a consequence, a lower rate of morbimortality can be reached in this population when submitted to hernial correction surgery^4.^

## CONCLUSION

Patients with cirrhosis submitted to abdominal wall hernia surgical correction have a high incidence of worsening their kidney function, with development of AKI in the first postoperative days. The patients who develop AKI in the postoperative phase have a higher rate of complications and higher mortality. Alongside worsening liver function, the risk factors for developing AKI in the postoperative phase are the presence of ascites, elevated baseline creatinine and the need for emergency surgery. 
